# A chromosome-level genome assembly of *Vanilla planifolia* uncovers the genomic architecture underlying partial endoreplication

**DOI:** 10.1186/s12864-026-12926-1

**Published:** 2026-05-13

**Authors:** Quentin Piet, Pascale Besse, Stéphanie Bocs, Olivier Bouchez, Mickael Bourge, Carine Charron, Charlotte Cravero, Amaury Dedours, Gaëtan Droc, Michel Dron, Michel Grisoni, Christophe Klopp, Fanny Lambert, William Marande, Hadrien Maté de Gérando, Nathalie Rodde, Marine Sallaberry, Sonja Siljak-Yakovlev, Marilyne Summo, Cyril Jourda

**Affiliations:** 1 UMR PVBMT, Université de la Réunion, Saint-Pierre, 97410 France; 2 UMR AGAP Institut, CIRAD, Montpellier, 34398 France; 3 UMR AGAP Institut, Univ Montpellier, CIRAD, INRAE, Institut Agro, Montpellier, 34398 France; 4 French Institute of Bioinformatics (IFB) - South Green Bioinformatics Platform, CIRAD, INRAE, IRD, Bioversity, Montpellier, 34398 France; 5 Genotoul, GeT-PlaGe, INRAE, Castanet-Tolosan, 31326 France; 6 Cytometry Facility, Imagerie-Gif, Institute for Integrative Biology of the Cell (I2BC), Université Paris-Saclay, CEA, CNRS, Gif-sur-Yvette, 91198 France; 7 UMR PVBMT, CIRAD, Saint-Pierre, 97410 France; 8 Centre National de Ressources Génomiques Végétales, INRAE, Castanet-Tolosan, 31326 France; 9 Eurovanille, Rue de Maresquel, Gouy-Saint-André, 62870 France; 10 Institute of Plant Sciences Paris-Saclay (IPS2), Université Paris-Saclay, CNRS, INRAE, Univ. Evry, Orsay, 91405 France; 11 UMR PVBMT, CIRAD, Tamatave, 501 Madagascar; 12 Plateforme Bioinformatique Genotoul BioinfoMics, UR875 Biométrie et Intelligence Artificielle, INRAE, 31320, Castanet-Tolosan, France; 13 Département Biotechnologie, V. Mane Fils, Le Bar Sur Loup, 06620 France; 14 Ecologie Société Evolution (ESE), Université Paris-Saclay, CNRS, AgroParisTech, Gif-sur-Yvette, 91190 France

**Keywords:** Vanilla planifolia, Genomics, Partial endoreplication, Chromosome-scale architecture, Long-read sequencing

## Abstract

**Background:**

Partial endoreplication is a prominent developmental feature and poses a significant challenge for whole genome assembly in orchids. This form of cell cycle results in highly unbalanced cell DNA content, with the highly endoreplicated (P) fraction being overrepresented in sequencing data compared to the non-endoreplicated (F) fraction.

**Results:**

Here, we report the first genome assembly of *Vanilla planifolia* into 16 chromosome pairs using axillary buds enriched in non-endoreplicated 2 C-nuclei (55%) as determined by flow cytometry. The assembly was generated using a hybrid approach combining PacBio HiFi sequencing and Omni-C scaffolding generated in this study, together with a GBS-SNP genetic map and Oxford Nanopore Technologies long-read data from the literature.

For the first time, we identified P and F regions within reconstructed chromosomes, representing 20.57% and 79.43% of the genome, respectively based on DNA sequencing data from three tissues with varying levels of endoreplicated nuclei. P regions were gene-rich and located at chromosome ends, whereas F regions were SSR-rich and located at central parts of chromosomes. Remarkably, 97.24% of SSRs were found in F regions, predominantly comprising the trinucleotide AAG/CTT motif, which may contribute to the absence of endoreplication in these regions.

Protein-encoding genes overrepresented in F regions were associated with negative regulation of flower development, mitotic cycle progression, cell division and histone modification.

**Conclusions:**

This accurate high-quality chromosome-scale V. planifolia genome assembly provides unprecedented insights into the structural and molecular characteristics of partial endoreplication in Orchids and represents a major step toward the characterization of this complex genome.

**Supplementary Information:**

The online version contains supplementary material available at 10.1186/s12864-026-12926-1.

## Background

Endoreplication is a common cell cycle variant in eukaryotes. It is a developmentally regulated process characterized by repeated S and G phases resulting from the exit from the mitotic cycle during the G2 phase or early mitotic stages, followed by a direct return to S phase. This leads to successive rounds of DNA replication without cell division, causing an exponential increase in nuclear DNA content [1]. Some orchid species exhibit a distinctive form of endoreplication, called partial endoreplication (PE), also known as progressively partial endoreplication [2–4] or strict partial endoreplication [5]. Unlike complete endoreplication (CE), cells undergoing PE replicate only a portion of their genomic sequence. Consequently, their nuclei exhibit less than a two-fold increase in DNA content after each replication cycle [6]. The nuclear DNA of PE species can be conceptualized as comprising two virtual fractions: a F fraction, which remains at the diploid level, and a P fraction, which undergoes endoreplication [5]. These fractions are considered to be fixed in organisms of a given species, independently of the developmental stage or organ, although the relative proportions of 2 C nuclei and of different classes of partially replicated nuclei vary among organs and tissues. This feature of PE, along with the fact that the resulting gametes are consistently haploid suggests a tightly regulated control of the endoreplication process. By restricting DNA replication to a subset of the genome, PE likely reduces the energetic and temporal costs associated with conventional endoreplication (CE), while preserving the functional advantages of increased nuclear DNA content. This selective replication may also limit the deleterious effects of repetitive DNA by excluding repeat-rich regions from amplification, while enhancing gene expression in endoreplicated fractions through gene dosage effects. Consequently, PE may support organ growth and metabolic activity, and contribute to plant performance under varying environmental conditions. These features suggest that PE plays a significant role in ecological adaptation and lineage diversification [3, 7, 8]. Selective genome amplification or rearrangement occurs in other biological systems. For example, polytene chromosomes result from repeated DNA replication without cell division and are found mostly in insects and also in mammals, protists and plants [9]. Plant polytene chromosomes are found restricted to some specific tissues in a few species [10]. Ciliates undergo programmed genome remodeling, amplifying somatic sequences while eliminating others [11] and some metazoans eliminate portions of their genome during development [12]. However, these patterns involve complete or variable endoreduplication rather than the systematically structured PE characteristic of Orchids.

To date, PE is specific to Orchids and the only form of endoreplication reported in the Vanilloideae and Orchidoideae subfamilies, whereas both PE and CE occur in Epidendroideae and Cypripedioideae and only CE has been documented in the basalmost subfamily Apostasioideae [3, 5, 13]. Among the Vanilloideae, *Vanilla planifolia* is a tropical orchid primarily propagated clonally through stem cuttings, due to limited natural pollination outside its native range. This has resulted in the widespread cultivation of a restricted number of genetically similar accessions (e.g., ‘Daphna’, ‘CR0040’). The species has undergone a relatively recent domestication process, with dissemination from its center of origin in Mesoamerica to major production areas such as Madagascar [2, 14]. It represents a model system to study PE with its P fraction encompassing 28.4% of its genomic sequence, which can be replicated up to 64 fold in cells showing the highest endoploidy level in this species [5]. Previous studies comparing the P and F fractions showed that the latter was mostly composed of repeats [4, 15]. More recently, 33% of *V. planifolia* genes were found in regions identified as non-endoreplicated based on sequencing depth comparisons among several tissues with different levels of PE [16]. From a genomics perspective, this uneven replication pattern inherent to PE introduces biases in sequencing coverage which challenges the *Vanilla* genome assembly. This may result in gaps, misassemblies, unanchored sequences, or missing regions. This highlights the need for a high-quality chromosome-level assembly to accurately resolve chromosomal organization and the distribution of the P and F fractions. In recent years, two chromosome-scale haplotype phased assemblies have been published [16, 17], each generated from different cultivars and organs (Daphna leaves and CR0040 nodes, respectively). Unfortunately, only 14 pairs of pseudomolecules were assembled whereas the basic number of chromosomes for the *Vanilla* genus is x = 16 [18] and two thirds of the sequence was either missing (Daphna) or unanchored (CR0040). Therefore, these assemblies did not allow for an analysis of structural characteristics including PE and the distribution of the F and P fractions along the chromosomes.

The *V. planifoli*a genome assembly completeness and contiguity are highly impacted by PE [16]. This is attributable to the underrepresentation of the F fraction in endoreplicated nuclei, leading to reduced sequencing depth, especially in tissues with high proportions of strongly endoreplicated nuclei, such as leaves, challenging the assembly of these repeat rich regions [5]. Thus, achieving sufficient coverage of this fraction should be possible by selecting tissues with low proportions of endoreplicated nuclei. In addition, the first genetic map for this species, composed of 16 linkage groups (LG), was recently assembled using a genotyping by sequencing (GBS) approach on a population obtained from self-fertilization of *V. planifolia* CR0040 [19]. The mapping of the flanking sequences of SNP used to construct the genetic map on the CR0040 and Daphna assemblies showed that chromosome 1 should be divided into three parts, thus resolving the mystery of the two missing chromosome pairs.

In the present study, we address the previously identified challenges of PE in genome assembly by combining flow cytometry, PacBio HiFi long-read sequencing, Omni-C scaffolding, and genetic map anchoring to generate a phased assembly of *V. planifolia*. This assembly is more complete than previous versions and successfully represents all 16 chromosome pairs. This high-quality genome and its annotation enabled us to delineate the chromosomal regions corresponding to the F and P fractions and to compare their composition in terms of genomic features.

## Methods

### Plant material

A cultivated *Vanilla planifolia* specimen from CIRAD, collected in La Reunion in 2003 by Michel Grisoni, was used in this study. CIRAD confirmed that this material is legally available for research purposes and is not subject to a Material Transfer Agreement. It was deposited under the voucher number CR-VA-00040 (also named “CR0040”) in the VATEL Biological Resource Center collection (Saint Pierre, La Réunion, France) on 10 March 2004. It complies with phytosanitary regulations (LOA permit n°03/RE2/01555). Formal taxonomic identification was made by the collector, and was further confirmed by molecular technologies including AFLP [2] and Genotyping-By-Sequencing [20]. It is maintained and publicly available by cuttings under shade-house and by in vitro culture established from axillary buds. The vitroplants were propagated by stem cuttings and grown in vitro in Murashige & Skoog medium under controlled conditions. The environment was maintained at 24–26 °C with a 12-hour light/dark cycle.

### Flow cytometry

The total nuclear DNA amount was assessed by flow cytometry as described in [21]. Leaves, roots and axillary buds from young *in vitro V. planifolia* (CR0040 cultivar) were chopped with a razor blade in a plastic Petri dish with 1 ml of Gif nuclei-isolation buffer (45 mM MgCl2, 30 mM sodium citrate, 60 mM MOPS, 1% (w/v) polyvinylpyrrolidone 10,000, pH 7.2) containing 0.2% (w/v) Triton X–100, supplemented with 5 mM sodium metabisulphite. The resulting suspension was filtered through 50-µm nylon mesh. Nuclei were stained with 5–10 µg/ml DAPI (or 50–100 µg/ml propidium iodide, PI) and kept 5 min at 4 °C. DNA content of 5,000–10,000 stained nuclei were determined for each sample using a cytometer (CytoFLEX S, Beckman Coulter. DAPI: Excitation 405 nm; emission through a 450/45 nm band-pass filter. PI: Excitation 561 nm; emission through a 610/20 nm band-pass filter). Nuclei were identified using a gate on Side-Scatter and DNA content-Area and the cytogram of DNA content-Area versus DNA content-Height signals served to select singlets, eliminating doublets and detecting any degradation. Frequencies of different ploidy levels were identified by their fluorescence intensity both in DAPI and PI to confirm ploidy levels. Endoreplication levels were determined from measurements obtained from five clonal vitropants of the cultivar CR0040 following the approach previously described [5]. Briefly, in tissues exhibiting PE, DNA fluorescence intensity profiles show a series of peaks that differ from those expected in conventional endoreplication (CE). Successive peaks follow a strict twofold progression in tissues with CE whereas in tissues with PE, the ratios between adjacent peaks are lower than two. This indicates that only a fraction of the genome is selectively replicated. These distinct peak patterns allow the endoreplication state of the cells to be accurately determined [2–5, 7, 8].

### High molecular weight (HMW) DNA extraction

Meristematic tissues from axillary buds were harvested from 44 CR0040 in vitro plantlets under binocular lenses. The harvesting process yielded approximately 180–190 mg of meristematic tissue, subsequently stored at -80 °C until DNA extraction. HMW DNA extraction was performed on the total amount of sampled axillary bud meristems using QIAGEN Genomic-tips 100/G kit (Qiagen, MD, USA). The tissues were mechanically grinded at 30 Hz for two intervals of 45 s each and extraction was performed following manufacturer’s instructions. Briefly, after 3 h of lysis and one centrifugation step, the DNA was immobilized on the column. After two washing steps, DNA was eluted from the column, then desalted and concentrated by isopropyl alcohol precipitation. A final wash in 70% ethanol was performed before resuspending the DNA in the buffer. Analyses of DNA quantity and quality were performed using NanoDrop and Qubit (Thermo Fisher Scientific, MA, USA). DNA integrity was also assessed using the Agilent FP-1002 Genomic DNA 165 kb on the Femto Pulse system (Agilent, CA, USA).

### DNA sequencing

Library preparation and sequencing were performed according to the manufacturer’s instructions “Procedure & Checklist – Preparing whole genome and metagenome libraries using SMRTbell^®^ prep kit 3.0”. At each step, DNA was quantified using the Qubit dsDNA HS Assay Kit (Life Technologies). DNA purity was tested using the nanodrop (Thermofisher) and size distribution and degradation assessed using the Femto pulse Genomic DNA 165 kb Kit (Agilent). Purification steps were performed using AMPure PB beads (PacBio) and SMRTbell cleanup beads (PacBio). A repair stage was performed on 10 µg of DNA using SMRTbell Damage Repair Kit SPV3 (PacBio) then sheared at 15 kb (speed V33) using the Megaruptor3 system (Diagenode). After a nuclease treatment, the library was size-selected using a 6 kb cutoff on the Pippin HT Size Selection system (Sage Science) with “0.75% Agarose, 6–10 kb High Pass, 75E” protocol. To obtain sufficient material for sequencing, this procedure was repeated on another 10 µg of DNA. The only difference is that the fragmentation was performed this time on the Megaruptor1 system at 20 kb. Libraries were sequenced using binding kit 3.2 by Adaptive Loading onto 12 SMRTcells on the PacBio Sequel II System instrument at 90 pM with a 2 hours pre-extension and a 30 hours movie.

### Evaluation of representativity bias in the sequencing data

PacBio HiFi CCS reads from *V. planifolia* CR0040 nodes [15] and newly generated PacBio HiFi CCS were aligned to the *V. planifolia* CR0040 V1 assembly [15] using Minimap2 v2.2.24 [22]. Mean read depths across 20 kb non-overlapping windows were calculated using mosdepth v0.3.3 [23] with ‘--fast_mode’ option. For each tissue, the mean depth of both chromosomal fraction and the unanchored sequences of the assembly was computed.

### Omni-C library preparation and sequencing

The Omni-C library (Dovetail Genomics^®^) was produced according to the manufacturer’s instructions. Briefly, close to 50 mg of frozen axillary bud meristems were grinded in liquid nitrogen and resuspended in PBS. Then, DNA was fixed with formaldehyde and digested using 0.3 µl of a nuclease enzyme mix. After binding 350 ng of the digested DNA to chromatin capture beads, a proximity ligation was performed and the crosslinks are reversed to produce the linked DNA. Finally, 54 ng of the linked DNA was used to produce an Illumina^®^ library. An initial run of 1 Gb data was produced to assess the library quality then 65X coverage data (150 Gb) was produced (paired-end reads 2 × 150 bp on an Illumina^®^ NovaSeq system).

### Genome assembly and pseudomolecules reconstruction

Axillary buds HiFi reads from *V. planifolia* CR0040, generated in the present study, were assembled using Hifiasm v0.19.5 [24], incorporating *V. planifolia* Daphna ONT long-read data [17] via the --ul option to improve the assembly contiguity. Contigs were checked for foreign organism contamination using NCBI’s FCS-GX v0.4.0 [25] and for mitochondrial and chloroplastic sequences using MitoHiFi v3.2 [26] with MitoFinder v1.4.1 [27]. Omni-C reads were then mapped to each haplotype using Juicer v1.5.7 [28] using default parameters. A single iteration of 3D de novo assembly v529ccf4 [29] with -r 0 parameter to limit automatic contig splitting was done before manual curation and scaffolding validation in Juicebox v2.17 [30]. The manual curation result was validated using additional information including alignment with *V. planifolia* CR0040 V1 scaffolds using D-GENIES [31], mapping of GBS-SNPs markers [19] using BWA-MEM2 v2.0 [32] and telomeric repeat locations using Seqtk v1.4-r130 [33] with the telomeric motif CCCTAAT and its reverse complement ATTAGGG [34]. The chromosome assembly completeness was evaluated using BUSCO v5.0.0 [35] with the viridiplantae_odb10 and liliopsida_odb10 lineages datasets. The k-mer analysis was performed with KAT v2.4.2 using the comp tool [36].

### Structural and functional annotation of genes

An automatic gene annotation was performed on contigs using the Nextflow version of EuGene Eukaryotic Pipeline v2.1.1 [37]. To train its internal statistical model, the *V. planifolia* Daphna proteome [17] and the *V. planifolia* CR0040 transcriptome were used in addition to protein evidence provided by the software. The transcriptome was produced using ten RNA-Seq datasets [16] mapped separately onto the first version of the *V. planifolia* CR0040 assembly with HISAT2 v2.2.1 [38] in an annotation guided fashion by providing splice sites and exons information from CR0040 V1 annotation. Exons and splice sites positions were first extracted from gene annotations using python scripts provided with the aligner and were used to build the reference genome index with the --ss and --exon options. Then, the ten RNA-Seq samples were aligned to the *V. planifolia* V2 assembly using HISAT2 with the --dta option. A first run of StringTie v2.0.3 [39] was executed by providing CR0040 V1 assembly gene annotation information. Finally, the ten tissue-specific transcriptomes were merged with ‘*stringtie --merge’* to obtain a non-redundant set of transcripts.

Functional annotation of protein-encoding genes was performed using Funcannotate [40] with default filtering parameters. Briefly, protein sequences were confronted to several biological databases including InterPro (InterProScan v5.67-99.0 [41]), KEGG (KofamScan v1.3.0 [42]), , HMM profiles (Gag retrotransposon and Gypsy-like retrotransposon), Repbase v23.12 [43], BUSCO (liliopsida_odb10), RefSeq proteomes of *Arabidopsis thaliana* (GCF_000001735.4), *Oryza sativa* (GCF_001433935.1) and *Phalaenopsis equestris* (GCF_001263595.1). UniProtKB/Swiss-Prot and TrEMBL databases were also queried, with TrEMBL entries filtered to retain only proteins with “protein existence” annotation score going from 1 to 3. Genic content completeness assessment was then carried out on this final set using BUSCO v5.0.0 with the viridiplantae_odb10 and liliopsida_odb10 lineages datasets.

Transposable elements (TEs) were detected, classified and localized using combined approaches. EDTA v2.2.0 [44], LTR_STRUC [45] and Inpactor2 [46] were run in parallel on the CR0040 genome sequence. PASTEClassifier [47] of REPET v3.0 [48] was used to filter on the Simple Sequence Repeats (SSRs, corresponding to sequences of 1–6 nt motifs repeated in tandem) coverage (≤ 50%) and to remove the Potential Host Genes. CD-HIT-EST [49] was used to remove identical TE between the three sets and to help during (i) the visual inspection of the longest TE dot plots using bl2seq [50] to remove those with a chimeric structure and (ii) the manual validation of PASTEClassifier results to remove redundancy results. TEannot from REPET v3.0 was run in order to keep only TE with at least one full length fragment and a combined total of ten fragments across both haplotypes. TEannot was used again with TE consensi identified in *Ophrys sphegodes* genome [51] in order to evaluate their conservation in *V. planifolia* CR0040 genome and to incorporate the most abundant ones in the final TE library. A last round of CD-HIT-EST clustering was done on the TE set enriched with RepeatModeler [52] and consensi previously identified in *V. planifolia* CR0040 genome [16] with RepeatScout [53] and cleaned using PASTEClassifier, TEannot and visual inspection of bl2seq dot plot of the longest consensi. The RepeatScout and RepeatModeler consensi were added to consolidate EDTA ones when their structure seemed valid (visual inspection of dotplots with Gepard [54]. At the end, *V. planifolia* CR0040 genome was annotated using TEannot and the non-redundant TE library newly produced.

SSRs (1–6 nt motifs) were identified with MISA v2.1 [55]. The minimum number of motif repetitions for a SSR to be considered was defined as ten for mononucleotides, seven for dinucleotides, six for trinucleotides and five for other repeat units (tetra- to hexanucleotides). SSRs separated by less than 100 nucleotides were considered as compound SSRs. Only those composed of motifs whose length was between 1 and 6 nucleotides were considered for statistics summary and further analyses.

### Synteny analysis

Synteny blocks and homologous gene pairs between haplotypes A and B of the CR0040 V2 assembly were computed by aligning identified protein sequences using a reciprocal best hit approach with NCBI BlastP and running MCScanX [56] with default parameters on the results.

### Detection and molecular characterization of P and F genomic fractions

The distinction between P and F chromosomal fractions was inferred from sequencing depth profiles. In species exhibiting PE, some genomic regions are not uniformly replicated during endocycle progression, leading to relative differences in DNA copy number across chromosomal regions. Normalized read depth is proportional to DNA copy number and so it can be used to infer genomic dosage variation, an approach originally developed for aneuploid plant cells [57]. Consequently, these dosage differences are directly reflected in sequencing depth variations. Regions assigned to the P fraction exhibit read depths proportional to the overall ploidy level, whereas F fraction regions are relatively underrepresented consistent with incomplete endoreplication. Comparative analysis of sequencing depth along the genome therefore enables the identification and quantification of these two chromosomal fractions. PacBio HIFI (axillary buds and nodes, CR0040 cultivar) and Illumina short reads (leaves, Daphna cultivar) were mapped on the final assembly with Minimap2 v2.2.24 [22] using the *map-hifi* preset and with BWA-MEM2 v2.0 [32], respectively. Secondary alignments were discarded using SAMTOOLS v1.14 [58] with the command ‘samtools view -bF 260 -q 1’. Depth values were computed at each genomic position with the command ‘samtools depth -@ 4 -aa -Q 1 -J -s’. For each tissue type, the distribution of the mapped reads along the chromosomes was segmented into 200 kb non-overlapping genomic windows. Each window was assigned a value corresponding to the median mapping depth calculated across all the positions within that window. The assembly was then partitioned into two fractions (P and F) using k-means clustering (k = 2) based on mapping results obtained exclusively from Illumina Daphna leaves reads, since they exhibited the greatest sequencing-depth contrast between high- and low-coverage regions. The clustering was applied to the normalized median sequencing depth values computed over 200 Kb windows across the genome. For each chromosome, depth values were first normalized by the chromosome’s mean sequencing depth prior to clustering. The midpoints between the centroids of P and F clusters, used as thresholds for window assignment to each fraction, ranged from 53.23x to 60.80x raw sequencing depth, reflecting chromosome-specific variation in coverage. Genes, TEs and SSRs densities were calculated in 200-kb genomic windows based on the annotation results using BEDOPS v2.4.39 [59] with the command ‘bedmap --echo --count’. Overlapping TEs were counted only once.

Gene ontology (GO) annotations of genes located in F and P fractions were used to perform a GO enrichment analysis for each fractions using the ‘enricher’ function from clusterProfiler v4.8.3 [60]. This function conducts an overrepresentation analysis based on a hypergeometric test comparing a gene set of interest to a background gene set. In our study, the background set comprised all genes associated with at least one GO term. P-values were adjusted using the Benjamini-Hochberg correction, with a significance threshold of 0.05.

## Results

### The first high-quality genome sequence of *Vanilla planifolia* anchored to all 16 chromosome pairs

**Fig. 1:**
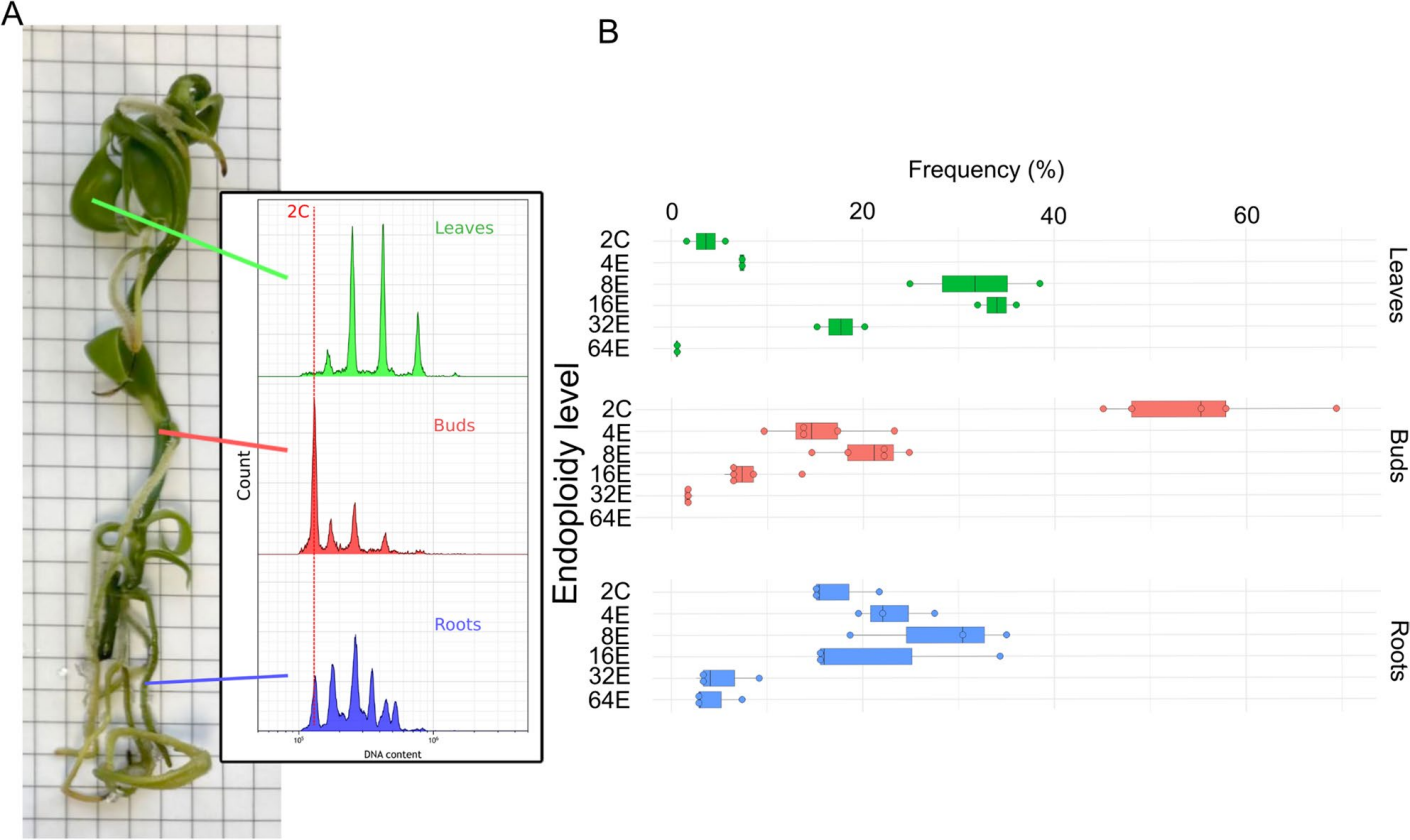
Flow cytometry DNA content distribution in leaves, axillary buds and roots of *Vanilla planifolia in vitro* plantlets. **A** The proportions of nuclei of various endoploidy states were measured. In the DNA content histograms from different tissues, the dotted line represents the expected position of the 2 C-peak. **B** Boxplot representation summarizing all measurements of DNA content made in nuclei isolated from the different tissues

Endoreplication rates in different tissues of in vitro plants (leaves, axillary buds and roots) were measured by flow cytometry. Figure 1 shows that axillary buds contained the highest proportion of 2 C-nuclei (55.12 ± 9.52%) compared to roots (17.36 ± 3.80%) and leaves (3.64 ± 2.85%). Based on these results and to limit the bias of representativeness of genomic fragments induced by PE, axillary buds from in vitro plants were selected for subsequent sequencing efforts.

The analysis of the primary alignments of the PacBio HiFi reads from axillary buds (8,240,084 reads) and from nodes (4,659,912 reads) against the CR0040 V1 assembly revealed that, on average, comparable sequencing depths were achieved across the 14 chromosome pairs of this previous assembly (Supplementary Table S1). Conversely, the previously unanchored scaffolds, associated with F regions, exhibited an increased average mapping depth when analysed using the newly generated axillary buds data. These results were reflected in the ratio between the average sequencing depth of chromosomes and unanchored regions, which decreased from 4.59 for nodes HIFI reads to 1.67 for axillary buds HIFI reads (Supplementary Table S1), indicating a more accurate representation of the F fraction of the *V. planifolia* genome in reads obtained from axillary buds DNA.

**Fig. 2:**
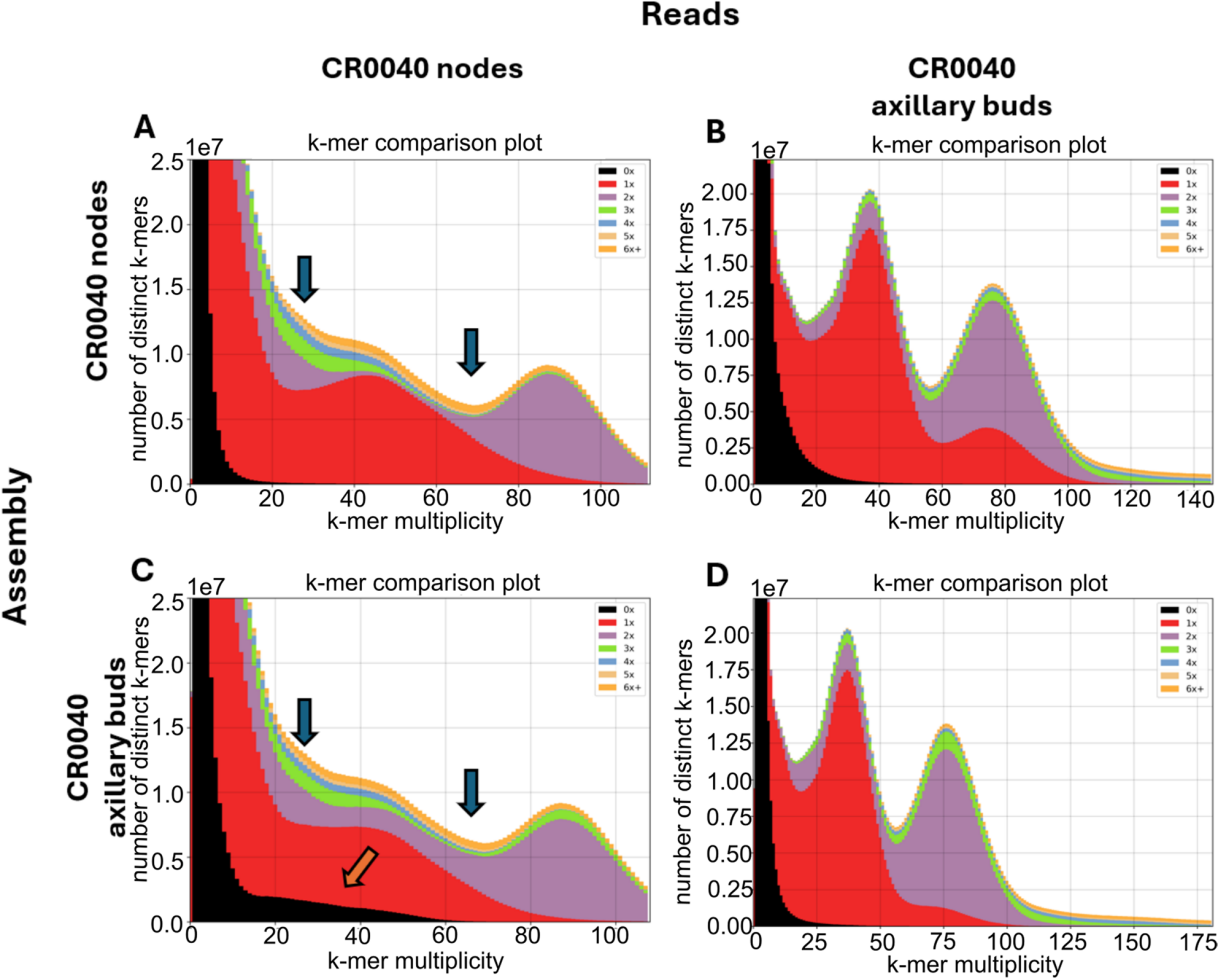
K-mers content comparison in sequencing reads and assemblies produced from DNA extracted from nodes and axillary buds of *V. planifolia* CR0040. Four spectra-cn plots are presented using k = 27: **A** CR0040 nodes reads versus CR0040 V1 assembly, (**B**) CR0040 axillary buds reads versus CR0040 V1 assembly, (**C**) CR0040 nodes reads versus CR0040 V2 assembly, (**D**) CR0040 axillary buds reads vers CR0040 V2 assembly. Each histogram represents the comparison between the 27-mers found in one of the two PacBio HiFi sequencing datasets versus the 27-mers found in one of the two CR0040 assemblies. The histogram presents for each coverage the number of distinct 27-mers (y-axis) present n times in the sequencing dataset (x-axis). Cumulative distributions are colored based on the number of times the corresponding 27-mers are found in the assembly (black : 0x, red: 1x, purple: 2x, green: 3x, blue: 4x, beige: 5x and orange: 6x). Blue arrows point towards k-mers showing intermediate depth levels compared to the expected heterozygous and homozygous distributions which correspond to the two peaks in each panel. The orange arrow points toward a missing fraction (as shown by the black-colored distribution) of k-mers in the present assembly but found in CR0040 nodes sequencing data

Secondly, a comparative analysis of the 27-mer distributions in sequences from axillary buds and nodes, as well as in the assembly, was performed (Fig. 2). HiFi reads obtained from nodes did not clearly reveal the two expected distributions (heterozygous and homozygous fractions, shown in red and purple respectively) for a diploid genome. Blue arrows in Fig. 2A and C show intermediate 27-mers depth levels between the expected heterozygous and homozygous distributions at ~ 30x and ~ 70x, which may result from a mixture of Gaussian distributions in the k-mers profiles of the P fraction of the genome at various endoploidy levels. Note that a typical diploid pattern should show two well separated peaks corresponding to heterozygous and homozygous distributions. Figure 2B and D show that the distributions corresponding to the expected heterozygous (single-copy 27-mers at ~ 37x) and homozygous (two copy 27-mers at ~ 77x) regions were better separated (two distinct peaks) in axillary buds reads than in nodes reads (Fig. 2A and C). Additionally, the area corresponding to low-depth k-mers (5x-15x) was considerably smaller in axillary bud reads than in node reads. Some 27-mers were overrepresented in copy number in both the heterozygous and homozygous distributions, with heterozygous 27-mers present in more than one copy and homozygous 27-mers in more than two copies in the assembly (Fig. 2A and B). The orange arrow in Fig. 2C shows that a fraction of 27-mers observed in the nodes sequencing data (V1 assembly) were absent from the V2 assembly.

**Fig. 3:**
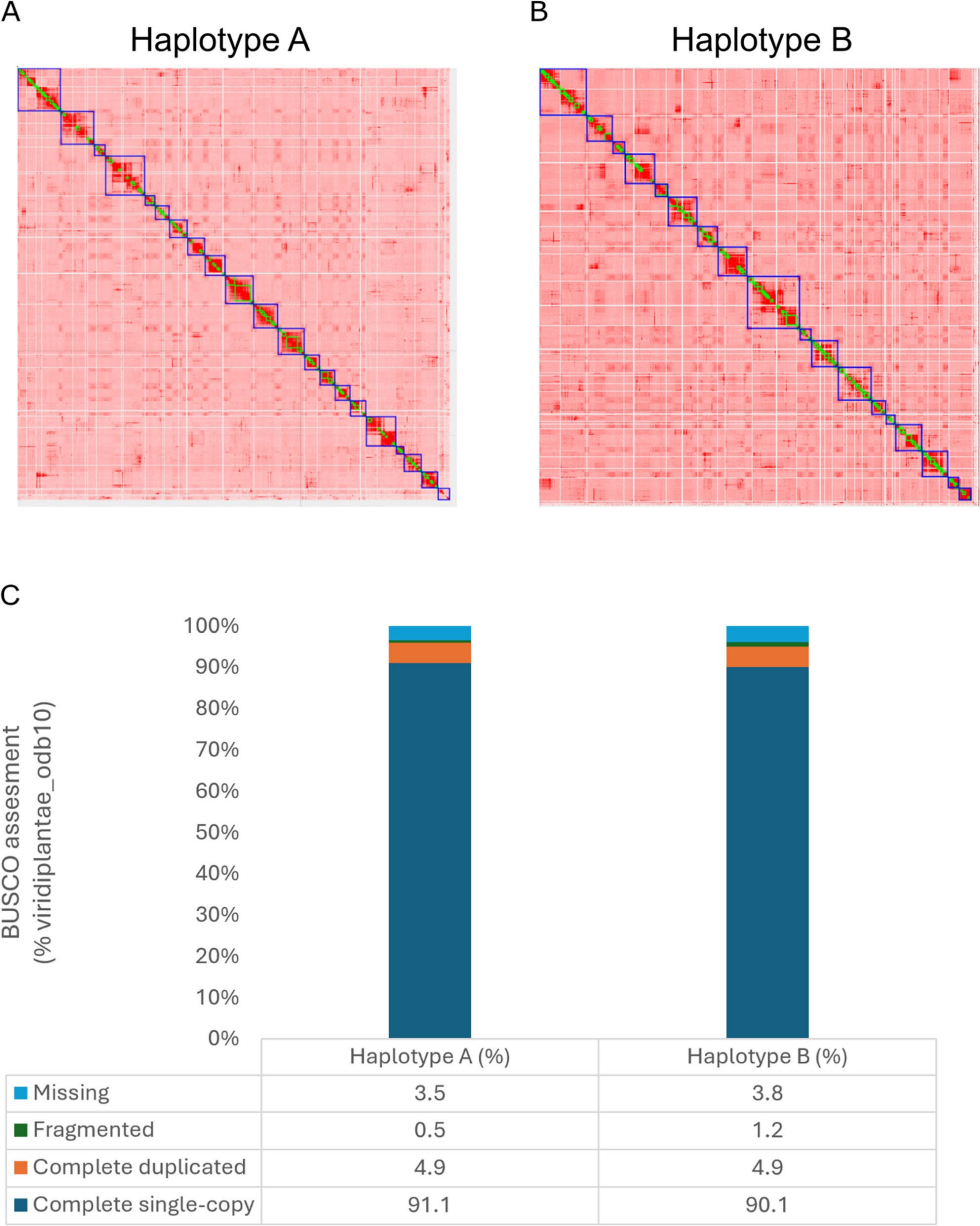
Omni-C contact maps from axillary buds of *V. planifolia* CR0040 haplotypes. **A** haplotype A and (**B**) haplotype B. The intensity of chromatin contacts between different genomic regions are indicated by the intensity of red in the Omni-C matrix. Blue frames: the 16 pairs of chromosomes (identified using the genetic map) and seven additional large unanchored scaffolds (positioned at the right end of the map); green frames: contigs from hifiasm assembly. **C** Benchmarking Universal Single-Copy Orthologs (BUSCO) assessment of the assembly completeness

GBS-SNPs markers combined with an Omni-C approach allowed to scaffold the 16 pairs of chromosomes (85.89% of the total assembly) from a total of 2,708 contigs with 1,794 and 914 contigs for haplotypes A and B respectively (Fig. 3A and B). The final assembly had a cumulative size of 3.53 Gb with a N50 of 105.76 MB organized into 16 pairs of chromosomes and 286 and 158 unanchored sequences for haplotype A and B, respectively (Table 1). The assembly completeness was estimated at 96% for haplotype A and 95% for haplotype B using the 425 conserved single-copy orthologous genes from the Viridiplantae (viridiplantae_odb10) BUSCO lineage. Additionally, the completeness was estimated at 91.3% and 90.2% using 3,236 conserved single-copy orthologous genes in Liliopsida for haplotype A and B, respectively (Fig. 3C). Following previously reported recommendations [61], pairs of chromosomes were labeled from chr1 to chr14 following previous nomenclatures [16, 17] except for the pair of chromosome 1 that was separated into three pairs of chromosomes following the information provided by the genetic map [19]. The largest portion was kept as chr1 while the two others were labeled chr15 and chr16. A total of 27 and 20 telomeric regions were identified in haplotype A and B, respectively (Supplementary Table S2). Five assembled chromosomes (chr1, 3, 4, 14 and 15) are still lacking the identification of a second telomere (combining haplotype A and B information).

**Table 1:** Final assembly and annotation statistics of the diploid *V. planifolia* CR0040 genome

Total assembly size (Gb)	3.53
Total sequences number	476
N50 (Mb)	105.76
Maximum sequence length (Mb)	191.23
Sequence in chromosomes (% sequence)	86.32
GC content (%)	31.87
Number of protein-encoding genes	63,872
BUSCO completeness	96.7
Total interspersed repeats content (% sequence)	32.98
Total SSR content (% sequence)	6.87

*Gb* Giga bases, *Mb* Mega bases

**Fig. 4:**
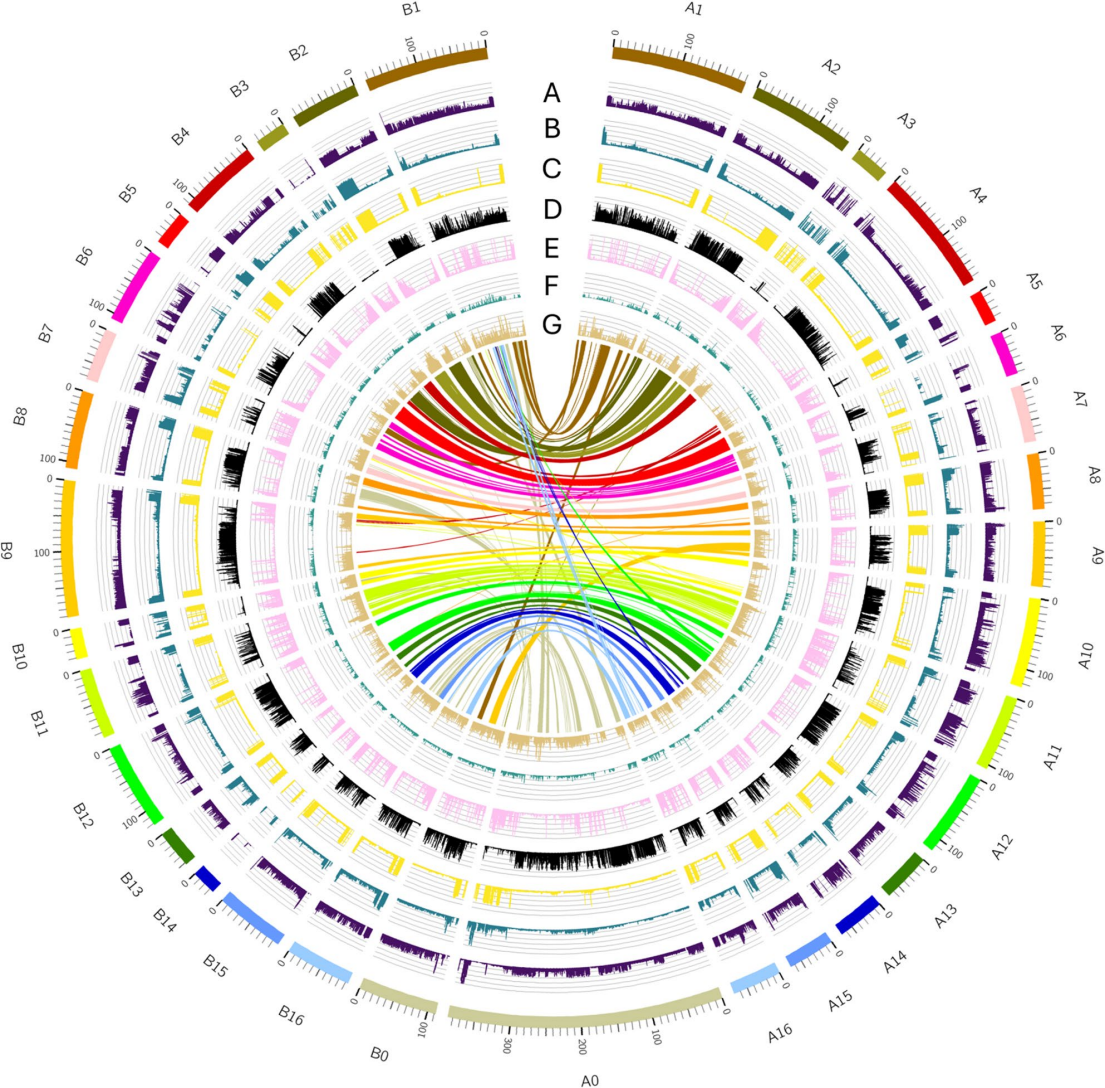
Circular representation of *V. planifolia* CR0040 phased assembly and its characteristics. The outer track represents the 16 chromosome pairs (with units in megabases). Unanchored sequences were concatenated and represented as two mosaic chromosomes (A0 and B0). Each colored link in the center represents a block of collinear genes between chromosomes (a total of 40,656 genes). Histograms in interior tracks represent genomic windows of 200 Kb (**A**) median values of mapping depth of CR0040 axillary buds PacBio HIFI reads, (**B**) median values of mapping depth of CR0040 nodes PacBio HIFI reads, (**C**) median values of mapping depth of Daphna leaves Illumina reads, (**D**) 1–6 nucleotides SSR density, (**E**) gene density, (**F**) DNA transposons density, (**G**) retrotransposons density

A total of 63,872 protein encoding genes were annotated and the 16 pairs of chromosomes contained the majority of these genes with 29,828 for haplotype A and 29,293 for haplotype B, representing 92.56% of the total annotated protein-encoding genes (Fig. 4 and Supplementary Table S3-S4). The remaining 4,751 protein-encoding genes were not anchored to the chromosomes.

The gene annotation completeness was estimated at around 93% for both haplotypes using the 425 conserved single-copy orthologous genes present in the Viridiplantae (virdiplantae_odb10) BUSCO lineage dataset (Supplementary Table S5). Collinearity analysis between both haplotypes showed that 40,656 genes were collinear. We identified a total of 2,078 repeats, including one satellite DNA, 1,228 transposable elements (TEs) and 849 unknown repeats (Supplementary Table S6) covering 39.85% of the genome. Excluding unknown repeats and ambiguously classified elements, LTR retrotransposons are the most abundant TE order (5.9% of the genomic sequence) with the predominant Gypsy superfamily (2.97%), followed by Copia (2.07%). LINE, SINE and other retrotransposons spanned 2.63%, 0.64% and 1.19% of the assembly, respectively. DNA transposon class II was rarer, representing 1.15% of the assembly. A total of 10,584,834 SSRs were identified, covering 6.87% of the diploid assembly sequence (Supplementary Table S4 and Supplementary Table S7). Low proportions of SSR were observed for chromosomes A3/B3, A5/B5 and B10. Additionally, the most prevalent class was trinucleotide SSR that comprised over 90% of the SSR identified for each haplotype (Supplementary Table S7). The motif AAG/CTT was the most common and represented 95.4% and 95.3% of the trinucleotides identified in haplotype A and B, respectively (Supplementary Table S8).

### Distribution of F and P genomic fractions along the 16 pairs of chromosomes

**Fig. 5:**
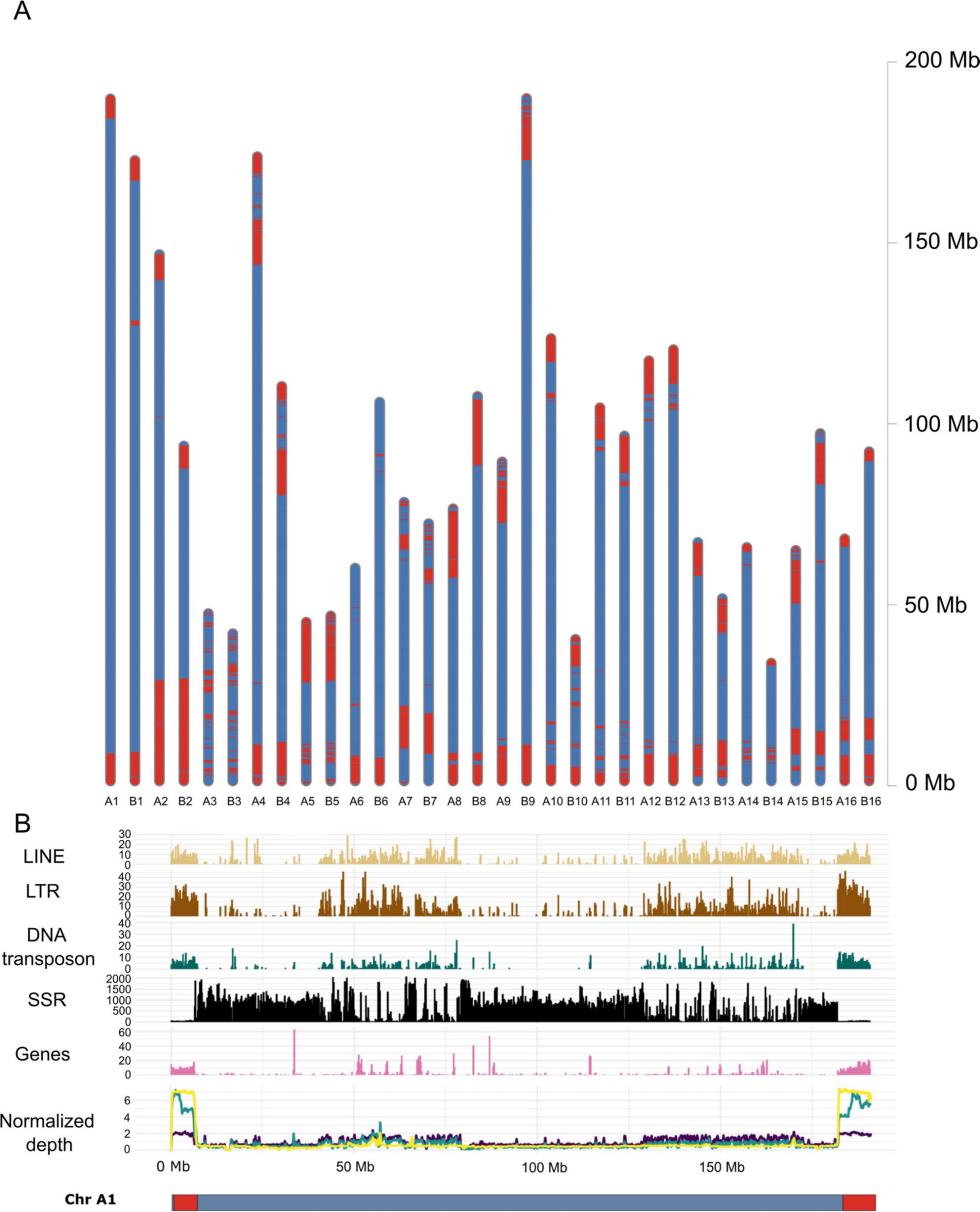
**A** Distribution of F and P regions along the 16 pairs of chromosomes and (**B**) detailed view of chromosome A1. F and P regions are indicated in blue and red respectively, along chromosomes represented by ideograms. The size bar is shown on the right in megabases (Mb). Distributions of LINE, LTR, DNA transposons, SSR and genes counts are indicated with beige, brown, green, black and pink histograms, respectively. Normalized sequencing depth from Daphna leaves (Illumina), CR0040 nodes (PacBio HIFI) and CR0040 axillary buds (PacBio HIFI) reads are indicated with yellow, green and purple lines, respectively. Size of chromosome A1 is indicated above the ideogram in Mb

Sequencing depth variations along the 16 chromosome pairs were analysed to identify F and P genomic fractions. The differential in sequencing depth between the two fractions varied by tissue (Figs. 4 and 5B), with the highest difference observed in Daphna leaves, followed by CR0040 nodes and CR0040 axillary buds. A conserved pattern was observed for most chromosomes, regardless tissue type. K-means clustering on the normalized depth median values per 200 Kb windows across the whole genome allowed the inference of F and P regions in the assembly. The normalized depth centroids for Daphna leaves median depth values were 0.26X for the F fraction and 4.28X for the P fraction, with a midpoint of 2.27X (corresponding to 59.21X in raw depth for the dataset). Low sequencing depth regions (F fraction) were predominantly located in the central part of chromosomes whereas high sequencing depth regions (P fraction) were mostly found at chromosome ends (Fig. 4) with the exception of the A3/B3 and A5/B5 pairs (Fig. 5A).

A similar pattern was observed in the Omni-C data which revealed a compartmentalization between large central regions and smaller terminal regions for all the chromosomes except for pairs A5/B5 and A3/B3 (Supplementary Fig. S1). The F fraction comprised a total of 2.41 Gb (1.23 Gb in haplotype A and 1.18 Gb in haplotype B), representing 79.43% of chromosomes (Supplementary Table S9). Alignment of the 16 pairs of chromosomes of the present assembly with the 14 pairs of the V1 assembly showed that the previously assembled chromosomes corresponded entirely to the P genomic fraction in the current assembly (Fig. 5A and Supplementary Fig. S2), indicating that missing or unanchored sequences in the previous assembly corresponded to the F fraction.

### Sequence composition of P and F genomic fractions

Gene and TE density were higher in the P fraction, whereas SSR were predominantly located in the F fraction, mostly in regions exhibiting lower sequencing depth across all three tissues studied (Figs. 4 and 5B). The different TE types (LINE, LTR, DNA transposons) appeared nested in similar locations along the chromosomes. Furthermore, TEs were found abundant in the P fraction together with genes, but were also found in the F fraction although only where the density of SSR is lower, as if there were differential preferential locations for SSR versus TEs (Fig. 5B). For each type of features, their distribution between F and P genomic fractions significantly deviated from a random expectation (Table 2).

**Table 2:** Preferential distribution of *V. planifolia* genomic features in the two genomic fractions

	Observed in *P* fraction	Observed in F fraction	Expected in *P* fraction	Expected in F fraction	*p*-value
Number of genes	27,421	31,877	12,199	47,098	***
Number of TE	383,842	728,146	228,769	883,218	***
Number of SSR	246,798	8,685,987	1,837,743	7,095,042	***

***, Chi-2 test p-value < 0.001

SSR were more abundant in the F fraction than randomly expected whereas genes and TEs (including each major TE category, Supplementary Fig. S3) were underrepresented in this fraction and more overrepresented in the P fraction relative to random expectation.

**Fig. 6:**
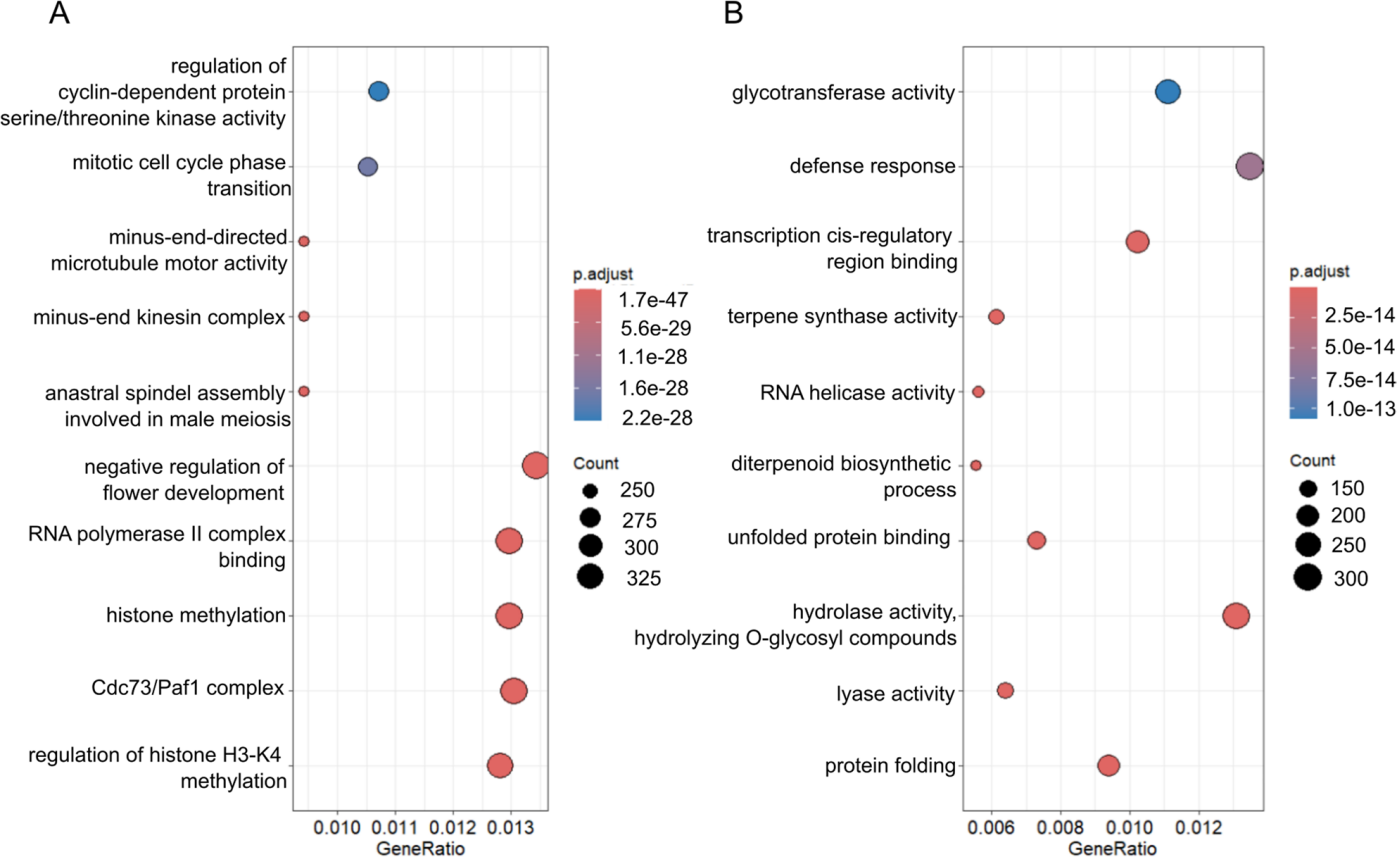
Functional enrichment analysis of (**A**) F regions and (**B**) P regions. The top ten significant GO enrichment results are illustrated for each fraction. Counts correspond to the number of genes associated with each GO term. P.adjust values were calculated using Benjamini-Hochberg correction

The GO enrichment analysis of the F and P fractions highlighted the preferential retention of specific functional categories within each fraction. Figure 6A shows the ten most significantly enriched GO terms in the F fraction, selected from a total of 58 overrepresented categories. They were related to histone methylation (GO:0016571, GO:0051569, GO:0016593, GO:0000993), cell cycle (GO:0044772), cytoskeletal activity (GO:0005872, GO:0009971, GO:0008569), negative flower regulation and regulation of cyclin-dependent kinase activity. The P fraction was enriched for a distinct set of GO categories, including those associated with defense response, protein folding and unfolded protein binding, terpenes and terpenoïds production, transcription and misc enzyme activities (Fig. 6B).

## Discussion

### Major improvements for the contiguity of *V. planifolia* assembly through PE impact consideration

The new *V. planifolia* assembly presented here encompasses over 86% of the genome size previously estimated by flow cytometry [16]. The mapping of telomeres across the 16 pairs of chromosomes revealed that five chromosomes were potentially lacking a second telomere. This observation may be explained by a combination of technical (missing telomeric regions in the current assembly) and biological (the occurrence of telocentric chromosomes in *V. planifolia*) factors. Previous metaphase observations indeed revealed that *V. planifolia* displays at least three telocentric chromosomes (corresponding to pairs 7, 11 and 13 on the karyotype in Fig. 2B in [16]) including one acrocentric pair carrying rDNA genes (pair 7). Fluorescence in situ hybridization (FISH) using telomeric probes could confirm the physical location of telomeres and detect telocentric chromosomes directly [62].

The discrepancy between the CR0040 V1 and Daphna assembly lengths was attributed to the impact of PE [16], inducing highly unbalanced DNA content within a given tissue and challenging the assembly of sequencing reads with highly unbalanced depths, particularly in low depth repetitive regions [63]. In this study, HiFi sequencing of DNA extracted from an alternative tissue (axillary buds) confirmed the impact of PE on sequencing depth. The mapping of axillary buds and nodes HiFi reads on CR0040 V1 assembly showed a reduction in the gap in sequencing depths between anchored and unanchored sequences for axillary buds in comparison to nodes. This indicated that the representation bias affecting the unanchored scaffolds was reduced in axillary buds, a tissue that is characterized in flow cytometry by lesser levels of endoreplicated nuclei compared to nodes. This observation was also confirmed by the mapping of DNA sequencing data from Daphna leaves (Illumina), CR0040 nodes (PacBio HIFI) and CR0040 axillary buds (PacBio HIFI) on the present CR0040 assembly. The sequencing depth difference between P and F fractions was substantially lower in axillary buds sequencing data than in nodes or leaves. The differential sequencing depth between the two fractions appeared to show a gradient across the three tissues that mirrored their respective endoreplication levels. Finally, the k-mers comparison of nodes and axillary buds showed an impact of tissue selection on k-mer content profiles, with observable k-mers of intermediate sequencing depths between the expected heterozygous and homozygous distributions for nodes.

### Chromosomal distribution and potential functional specialization of F and P genomic fractions

Across both fractions along the 16 pairs of chromosomes, the first type (P fraction) corresponded to high-depth regions located mainly at chromosome ends and enriched in genes and TEs. The second type (F fraction) corresponded to low-depth regions located in central chromosome regions and enriched in microsatellite sequences. This pattern is consistent with observations in other eukaryotic species, where SSR are reported to be more abundant in non-coding regions [64]. This compartmentalization of the two fractions was also observed in the chromosome contact maps derived from Omni-C data (Supplementary Fig. S1), suggesting a heterochromatin-euchromatin organization, with central regions composed of highly repetitive heterochromatin and peripheral regions consisting of gene-rich euchromatin. The F fraction represented 79.43% of the chromosome sequences which is close to the value previously estimated by flow cytometry (69.5% ±3.2%) for CR0040 [16]. Our strategy efficiently revealed, for the first time, the broad distribution of PE regions along the chromosomes, although 14% of the estimated genome size remains missing and some scaffolds have yet to be anchored to chromosomes.

A total of 59,121 genes (92.56% of total predicted genes) were anchored on the 16 pairs of chromosomes with preferential positions at the chromosome ends (P fraction). The P fraction was enriched in genes, but this does not mean that genes were totally absent from the F fraction. There were, in fact, more genes annotated in the P fraction than in the F one, but this number was rather low compared to the size of the F fraction and some GO categories were significantly found overrepresented in F regions. The top ten GO categories are related to three main biological processes which are histone modification, cell cycle and cell division and regulation of floral development. Based on GO terms, many gene families involved in epigenetic mechanisms have been retrieved in these F regions. These have a central role in heterochromatic gene silencing, either constitutive or transient. Histone methylation is known to play a fundamental role in epigenetic regulation of the expression of numerous genes in plant responses to abiotic stresses [65]. The mechanisms involved in the regulation of flowering have been particularly studied in *Arabidopsis*, in perennial and annual species [66–68]. The FLOWERING LOCUS C (FLC), or PEP1 in perennial, encodes a central repressor of the entire molecular cascade regulating the transition from a vegetative state to a reproductive state, and its strong expression from germination blocks all flowering. Thus, it maintains the plant in a vegetative state as long as it is not itself inhibited by combined exposure to low temperature and short photoperiods which will thus allow the activation by the circadian cycle of the flowering trigger factor. Epigenetic modifications such as triggering of chromatin modifications allow for a stably transcription repression of FLC or PEP1. The enrichment of genes with functions related to flowering regulation in F regions of *V. planifolia* could suggest that these regions correspond to facultative heterochromatin, i.e. a temporarily repressive chromatin state, in order to temporally regulate the expression of genes responsible for these specific cellular programs key for plant reproduction [69–71]. The enrichment of defense-related genes in the P fraction suggests a potential functional bias toward biologically active regions. In plants, endoreplication has been associated with increased transcriptional capacity and gene amplification [1, 72], as well as with responses to environmental and stress conditions [73, 74], which could support the preferential amplification of genes involved in adaptive processes, including defense. However, to our knowledge, no direct evidence currently demonstrates a specific association between the P fraction and immune defense in *V. planifolia*, and this hypothesis remains to be further investigated.

The annotation strategy integrates a combination of transcriptomic and proteomic evidence, as well as ab initio methods. Recent deep learning-based tools such as Helixer [75] and ANNEVO [76] have demonstrated improved performance in gene prediction across multiple species. Incorporating such approaches in future analyses could further refine gene models and provide a more comprehensive view of the gene content in both P and F regions.

### Chromatin type as a marker of PE chromosome landscape

*V. planifolia* chromosomes possess important portions of telomeric and pericentromeric unspecific heterochromatin resulting in many chromocenters visible in interphase nuclei after DAPI and Orcein staining [16]. Hoechst staining revealed that A-T rich heterochromatin regions were more numerous than G-C rich regions [16]. The telomeric A-T rich heterochromatin could correspond to the *V. planifolia* telomeric repeats (ATTAGGG) whereas pericentromeric A-T rich heterochromatin could correspond to the high density AAG/CTT microsatellites observed in the central part of most chromosomes (F fraction). This also confirms the first hypothesis raised in *Vanilla* [77] and later in orchids [4, 15] stating that the F fraction likely corresponds to heterochromatin regions rich in repeated sequences It was also shown that genome size expansion in PE and CE orchids was due to repeated element proliferation, which was limited to the F fraction for PE species [7]. Our results show that this repeated element proliferation in *V. planifolia* concerns mainly the AAG/CTT repeats and some TEs (65.48%) located in the F fraction. Finally, our results also demonstrated that the P fraction corresponds to gene-rich euchromatin regions in the *V. planifolia* genome. This is consistent with nuclei imaging experiments showing increased transcriptional activity with increased level of *Vanilla* endoreplicated nuclei, therefore confirming that the endoreplicated fraction was transcribed [5]. Interestingly, their analysis of base composition showed a trend with a possible increase in chromatin content (G-C rich) compared to A-T rich regions which tended to stay fixed [5].

### AAG/CTT repeats are prone to expansion in *V. planifolia* genome and may be potential drivers of endoreplication suppression

SSR abundance in *V. planifolia* (6.87% of the genome) was higher than in various land plants [78, 79]. *V. planifolia* presents a very specific microsatellite motif AAG/CTT which has invaded the F regions of the chromosomes. This motif represents over 90% of the total SSR and 95% of the trinucleotide microsatellites identified in the genome. This non-random genomic distribution and abundance indicates a major functional role. Most SSR are neutral, but some are well known for their functional significance [64]. SSRs, when able to adopt secondary structures, can impede DNA replication and promote chromosomal fragility, genomic instability, and DNA breakage [80, 81]. No such tri-nucleotide pattern is, to our knowledge, known in plants. In plant species, trinucleotide repeats, including AAG/CTT, are only found more frequent when considering coding regions [1, 82, 83], as in the case of human trinucleotide repeats [84]. and the motif is never unique. Such sequences known as TRS (Triplet Repeat Sequences) are well studied in the human genome. The expansion of TRS has been correlated with about 50 human genetic disorders (triplet repeat expansion diseases (TREDs)) such as fragile X syndrome and Huntington’s disease [85–88]. Most early known TRS were CTG/CAG and CGG/CCG, but the intronic AAG/CTT was identified later as responsible for Friedreich’s ataxia (FRDA) [86, 87, 89, 90]. These TRS are known to adopt atypical secondary structures inducing genetic instability through errors in DNA replication [85]. Indeed, biophysical and biochemical studies have revealed that five (CGG, CCG, CAG, CTG and AAG) of the six TRS that are associated with human diseases can form stable hairpin structures and are therefore likely to undergo expansion through replication slippage, responsible for the observed diseases [86]. This might also therefore explain the incredible expansion of the AAG/CTT sequence observed in the *V. planifolia* genome.

In addition to hairpin structures, some of these sequences (AAG/CTT and AGG/CCT) can also form other non-B DNA secondary structures such as intramolecular triplexes (triple-stranded DNA) in vitro [86, 88, 89]. The triplex structure can stop DNA synthesis [87] and generate pausings of DNA polymerase in vitro [89]. Finally, AAG/CTT repeats can also form secondary R-loops (RNA: DNA structures) which were suggested to be responsible together with triplexes for the reduction of abundance of mature X25 mRNA in individuals with FRDA [88, 89]. Additionally, in *Drosophila*, heterochromatic satellite repeats have been presented as a barrier to DNA replication and correspond to underreplicated regions in endopolyploid cells, leading to formation of polytene chromosomes [91]. In a plant genome, SSR‑rich regions can act as obstacles to DNA replication. Consequently, the numerous AAG/CTT repeats identified in the non-endoreplicated F fraction of *V. planifolia* may function as replication barriers. They could be directly responsible via triplex structure formation for the lack of endoreplication of the fraction containing them. It would be interesting to investigate whether similar repeats are present in the F fraction of other PE orchids.

## Conclusions

This study reports the first chromosome-scale genome assembly of *V. planifolia*, resolved into 16 chromosome pairs, representing the most complete assembly available to date. This resource enabled new insights into the structural organization of PE and the genomic composition of its F and P fractions along the chromosomes, confirming the high gene density characteristic of the P fraction and revealing the SSR-rich nature of the F fraction. The availability of this improved reference genome paves the way to future physical characterization based on chromosome FISH (fluorescent in situ hybridization), by designing and using synthetic oligonucleotide (oligo) probes [92, 93] specific for the P fraction on each chromosome, and by using a AAG/CTT probe to confirm the F fraction localization in situ. In addition, this work provides practical guidelines to overcome the challenges imposed by PE during genome sequencing and assembly, notably through the use of flow cytometry to select appropriate tissues. Finally, the assembled genome, together with the delineation of endoreplicated and non-endoreplicated regions, establishes a valuable foundation for future investigations of PE in orchid genomes.

## Supplementary Information

Supplementary Material 1.

## Data Availability

The raw sequencing data generated in this study, including PacBio HiFi CCS and Omni-C reads, are available in the NCBI Sequence Read Archive under BioProject PRJNA1254293. The assemblies and associated datasets have been deposited in NCBI under BioProjects PRJNA1254293 (haplotype A) and PRJNA1254300 (haplotype B). In addition, the assemblies, gene annotations and predicted protein sequences are also publicly accessible via the Vanilla Genome Hub (https://vanilla-genome-hub.cirad.fr/downloads).

## Abbreviations

Bp: Basepairs
BUSCO: Benchmarking Universal Single-Copy Orthologs
CCS: Circular consensus reads
Gb: Gigabyte bases
Mb: Million bases
NCBI: National Center for Biotechnology Information
PE: Partial endoreplication
SSR: Simple sequence repeats
TE: Transposable element(s)
