# Supplementary material for: A chromosome-level genome assembly of Vanilla planifolia uncovers the genomic architecture underlying partial endoreplication
Source: BMC Genomics. 2026 May 13;27:589. doi: 10.1186/s12864-026-12926-1 (PMC13340410; doi:10.1186/s12864-026-12926-1)
Supplement: Supplementary file 1 — Supplementary Material 1. [file 12864_2026_12926_MOESM1_ESM.docx]

**SUPPLEMENTAL INFORMATION**

**Table S1.** Comparison of the anchored and unanchored *V. planifolia* CR0040 V1 assembly regions sequencing depths computed from primary alignments of CR0040 nodes HIFI reads and CR0040 axillary buds HIFI reads

|  | CR0040 V1 nodes HIFI reads | CR0040 V2 axillary buds HIFI reads |
| --- | --- | --- |
| Chr hap A average depth | 47.11 | 48.33 |
| Chr hap B average depth | 38.32 | 29.50 |
| Chr average depth | 42.92 | 39.34 |
| Unanchored hap A average depth | 10.40 | 28.10 |
| Unanchored hap B average depth | 8.75 | 20.92 |
| Unanchored average depth | 9.34 | 23.48 |
| Whole genome average depth | 18.99 | 28.04 |
| Ratio chr/unanchored | 4.59 | 1.67 |

Chr, chromosome; hap, haplotype

**Table S2.** Structural metrics of *V. planifolia* CR0040 chromosomes

|  | Haplotype A | |  | Haplotype B | |
| --- | --- | --- | --- | --- | --- |
| Chromosome | Length (bp) | Number of telomeres |  | Length (bp) | Number of Telomeres |
| 1 | 191,104,892 | 1 |  | 174,117,371 | 1 |
| 2 | 148,081,169 | 2 |  | 95,179,616 | 1 |
| 3 | 48,875,526 | 1 |  | 43,309,348 | 1 |
| 4 | 175,139,888 | 2 |  | 111,654,360 | 0 |
| 5 | 46,485,078 | 1 |  | 48,227,611 | 1 |
| 6 | 61,432,256 | 1 |  | 107,275,398 | 2 |
| 7 | 79,653,178 | 1 |  | 73,734,762 | 2 |
| 8 | 77,831,392 | 1 |  | 108,885,162 | 2 |
| 9 | 90,794,993 | 2 |  | 191,227,019 | 2 |
| 10 | 124,883,241 | 2 |  | 41,754,004 | 2 |
| 11 | 105,755,710 | 2 |  | 97,949,161 | 2 |
| 12 | 118,754,671 | 2 |  | 121,808,810 | 1 |
| 13 | 68,534,059 | 2 |  | 53,003,415 | 0 |
| 14 | 67,152,216 | 1 |  | 35,193,778 | 0 |
| 15 | 66,345,247 | 1 |  | 98,609,927 | 1 |
| 16 | 69,527,048 | 2 |  | 93,600,593 | 1 |
| 1-16 | 1,540,350,564 | 24 |  | 1,495,530,335 | 19 |
| Unanchored | 387,402,839 | 3 |  | 111,352,455 | 1 |
| Total | 1,927,753,403 | 27 |  | 1,606,882,790 | 20 |

Bp, base pairs

**Table S3.** Summary statistics of protein coding genes per haplotype

|  | Haplotype A | Haplotype B |
| --- | --- | --- |
| Number of protein-coding genes | 33,525 | 30,347 |
| Number of exons | 157,116 | 149,227 |
| Number of single-exon genes | 8,236 | 6,828 |
| Mean gene length (bp) | 6,960 | 7,524 |
| Mean cds length (bp) | 1,062 | 1,093 |
| Mean exon length (bp) | 287 | 285 |
| Mean protein length (aa) | 354 | 364 |

Bp, base pairs; cds, coding sequence; aa, amino acid

**Table S4.** Distribution of protein-encoding genes, TEs and SSR (1-6 nucleotides) per sequences in each haplotype of CR0040 assembly

|  | Haplotype A | | |  | Haplotype B | | |
| --- | --- | --- | --- | --- | --- | --- | --- |
| Chromosome | number of genes | TE  (% bp) | SSR  (% bp) |  | number of genes | TE  (% bp) | SSR  (% bp) |
| 1 | 1,875 | 23.60 | 7.61 |  | 2,486 | 30.88 | 6.55 |
| 2 | 1,784 | 24.44 | 11.04 |  | 1,641 | 29.40 | 8.64 |
| 3 | 1,735 | 54.82 | 1.11 |  | 1,515 | 53.14 | 0.48 |
| 4 | 1,958 | 26.10 | 8.83 |  | 1,755 | 30.92 | 8.70 |
| 5 | 2,047 | 38.26 | 1.30 |  | 2,179 | 38.18 | 1.27 |
| 6 | 1,576 | 42.50 | 3.68 |  | 1,618 | 31.84 | 6.01 |
| 7 | 1,422 | 30.63 | 6.29 |  | 2,012 | 48.23 | 2.58 |
| 8 | 1,506 | 27.65 | 9.33 |  | 1,574 | 29.59 | 8.80 |
| 9 | 1,776 | 30.95 | 6.77 |  | 2,443 | 29.06 | 7.72 |
| 10 | 1,874 | 28.13 | 8.94 |  | 1,517 | 48.65 | 0.61 |
| 11 | 3,384 | 55.60 | 2.94 |  | 2,696 | 51.96 | 3.64 |
| 12 | 1,729 | 23.56 | 10.19 |  | 1,680 | 23.93 | 10.02 |
| 13 | 1,090 | 27.11 | 7.56 |  | 1,107 | 30.21 | 6.80 |
| 14 | 2,084 | 45.07 | 4.97 |  | 960 | 35.11 | 4.68 |
| 15 | 1,820 | 24.52 | 5.76 |  | 2,415 | 34.84 | 4.57 |
| 16 | 2,168 | 37.06 | 5.64 |  | 1,695 | 31.70 | 5.67 |
| 1-16 | 29,828 | 31.45 | 7.19 |  | 29,293 | 33.97 | 6.29 |
| unanchored | 3,697 | 30.92 | 8.30 |  | 1,054 | 40.28 | 5.37 |
| Total | 33,525 | 31.35 | 7.42 |  | 30,347 | 34.40 | 6.22 |

Bp, base pairs; TE, Transposable elements; SSR, Simple sequence repeats (1-6 bp)

**Table S5.** Completeness assessment of *V. planifolia* CR0040 final annotation using BUSCO

|  | Haplotype A (nb) | Haplotype A (%) | Haplotype B (nb) | Haplotype B (%) | Haplotypes A + B (nb) | Haplotypes A + B (%) |
| --- | --- | --- | --- | --- | --- | --- |
| Genes in viridiplantae_odb10 dataset | 425 | 100 | 425 | 100 | 425 | 100 |
| Complete single-copy | 378 | 88.9 | 379 | 89.2 | 26 | 6.1 |
| Complete duplicated | 21 | 4.9 | 17 | 4.0 | 381 | 89.6 |
| Fragmented | 13 | 3.1 | 13 | 3.1 | 9 | 2.1 |
| Missing | 13 | 3.1 | 16 | 3.7 | 9 | 2.2 |
| Genes in liliopsida_odb10 | 3,236 | 100 | 3,236 | 100 | 3,236 | 100 |
| Complete single-copy | 2,720 | 84.1 | 2,684 | 82.9 | 200 | 6.2 |
| Complete duplicated | 147 | 4.5 | 154 | 4.8 | 2739 | 84.6 |
| Fragmented | 150 | 4.6 | 150 | 4.6 | 116 | 3.6 |
| Missing | 219 | 6.8 | 248 | 7.7 | 181 | 5.6 |

Nb, number

**Table S6.** Repeat annotation statistics

| Repeat category | REPET Wcode | Number of sequences in the library | Cumulative size of fragments (percentage of  haplotypes A + B) | Number of fragments (haplotypes A +B) |
| --- | --- | --- | --- | --- |
| LTR Copia | RLC | 112 | 2.07 | 87,844 |
| LTR Gypsy | RLG | 173 | 2.97 | 124,418 |
| Unclassified LTR | RLX | 2 | 0.86 | 18,632 |
| Penelope | RPX | 3 | 0.01 | 661 |
| LINE | RIX | 106 | 2.63 | 106,165 |
| Unclassified retrotransposons | RXX | 29 | 0.82 | 38,341 |
| SINE | RSX | 9 | 0.64 | 21,006 |
| LARD | RXX-LARD | 11 | 0.13 | 7,165 |
| TRIM | RXX-TRIM | 16 | 0.23 | 8,588 |
| TIR DNA transposons | DTX | 181 | 0.70 | 50,638 |
| Helitron | DHX | 5 | 0.02 | 1,904 |
| Maverick | DMX | 1 | 0.01 | 623 |
| Unclassified DNA transposons | DXX | 7 | 0.40 | 13,837 |
| MITE | DXX-MITE | 34 | 0.02 | 4,029 |
| Ambigous TE | confused | 539 | 7.61 | 263,604 |
| Satellite DNA | DNA-sat | 1 | 6.87 | 83,086 |
| Unknown repeat | XXX | 849 | 13.86 | 750,557 |
| Total | | 2,078 | 39.85 | 1,581,098 |

**Table S7.** Proportions of motif lengths in the SSR found in CR0040 V2 assembly relative to the total number of SSR.

|  | Haplotype A | |  | Haplotype B | |  | Haplotypes A + B | |
| --- | --- | --- | --- | --- | --- | --- | --- | --- |
| SSR motif length | number | proportion (%) |  | number | proportion (%) |  | number | proportion (%) |
| Mono | 170,182 | 2.7 |  | 153,994 | 3.5 |  | 324,176 | 3.0 |
| Di | 266,262 | 4.3 |  | 245,710 | 5.6 |  | 511,972 | 4.8 |
| Tri | 5,741,085 | 92.2 |  | 3,946,862 | 90.1 |  | 9,687,947 | 91.3 |
| Tetra | 5,699 | 0.1 |  | 4,790 | 0.1 |  | 10,489 | 0.1 |
| Penta | 34,430 | 0.6 |  | 22,621 | 0.5 |  | 57,051 | 0.5 |
| Hexa | 9,232 | 0.1 |  | 6,218 | 0.1 |  | 15,450 | 0.1 |
| total | 6,226,890 | 100 |  | 4,380,195 | 100 |  | 10,607,085 | 100 |

**Table S8.** Proportions of trinucleotide motifs in CR0040 V2 haplotypes A and B relative to the number of trinucleotide SSR.

|  | Haplotype A | |  | Haplotype B | |
| --- | --- | --- | --- | --- | --- |
|  | number | proportion (%) |  | number | proportion (%) |
| AAG/CTT | 5,475,405 | 95.4 |  | 3,762,985 | 95.3 |
| AAT/ATT | 94,522 | 1.6 |  | 65,600 | 1.7 |
| AGG/CCT | 59,635 | 1 |  | 40,966 | 1 |
| ATC/ATG | 70,217 | 1.2 |  | 48,050 | 1.2 |

**Table S9.** Sizes of F and P fraction in haplotype A and haplotype B.

|  | Haplotype A | |  | Haplotype B | |
| --- | --- | --- | --- | --- | --- |
| Chromosome | F fraction (Mb) | P fraction (Mb) |  | F fraction (Mb) | P fraction (Mb) |
| 1 | 175.80 | 15.30 |  | 157.11 | 17.00 |
| 2 | 111.88 | 36.20 |  | 59.40 | 35.77 |
| 3 | 37.20 | 11.67 |  | 32.00 | 11.30 |
| 4 | 144.80 | 30.33 |  | 81.25 | 30.40 |
| 5 | 24.48 | 22.00 |  | 26.62 | 21.60 |
| 6 | 52.83 | 8.60 |  | 98.87 | 8.40 |
| 7 | 61.25 | 18.40 |  | 55.33 | 18.40 |
| 8 | 51.03 | 26.80 |  | 82.48 | 26.40 |
| 9 | 65.80 | 24.99 |  | 165.42 | 25.80 |
| 10 | 108.00 | 16.88 |  | 26.20 | 15.55 |
| 11 | 88.60 | 17.15 |  | 80.00 | 17.94 |
| 12 | 96.60 | 22.15 |  | 100.60 | 21.20 |
| 13 | 50.60 | 17.93 |  | 34.40 | 18.60 |
| 14 | 61.95 | 5.20 |  | 30.79 | 4.40 |
| 15 | 44.34 | 22.00 |  | 78.20 | 20.40 |
| 16 | 51.60 | 17.92 |  | 75.80 | 17.80 |
| 1-16 | 1226.78 | 313.57 |  | 1184.42 | 311 |

Mb, Megabases


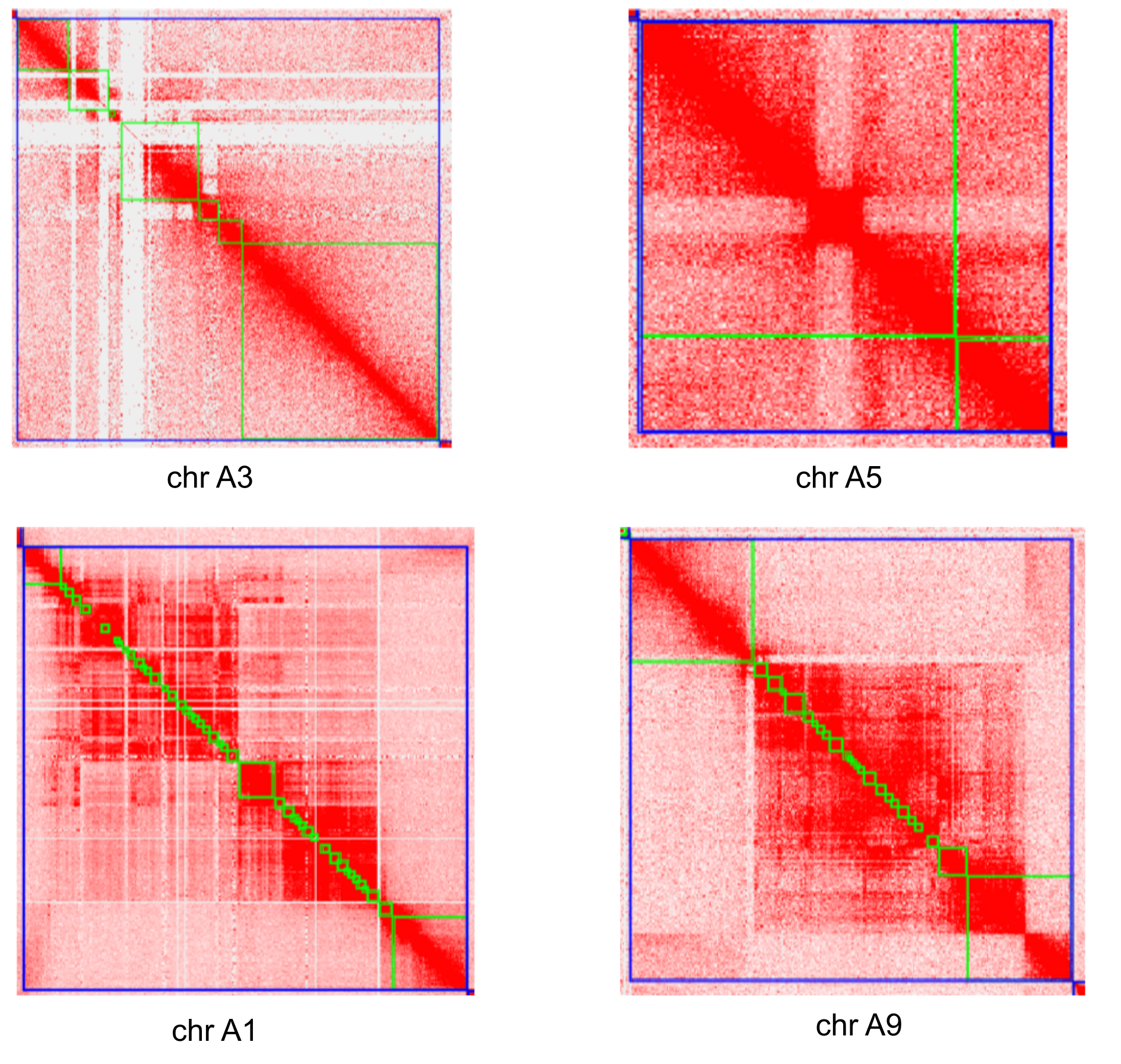


**Figure S1.** Hi-C contact map of chromosomes A3, B5, A1 and A9 displayed with Knight-Ruiz normalization.

The intensity of chromatin contacts between different genomic regions are indicated by the intensity of red in the Hi-C matrix. Blue frames: chromosomes; green frames: contigs from hifiasm assembly. Chromosomes A3 and A5 show a different pattern than the rest of the chromosomes (illustrated by A1 and A9).


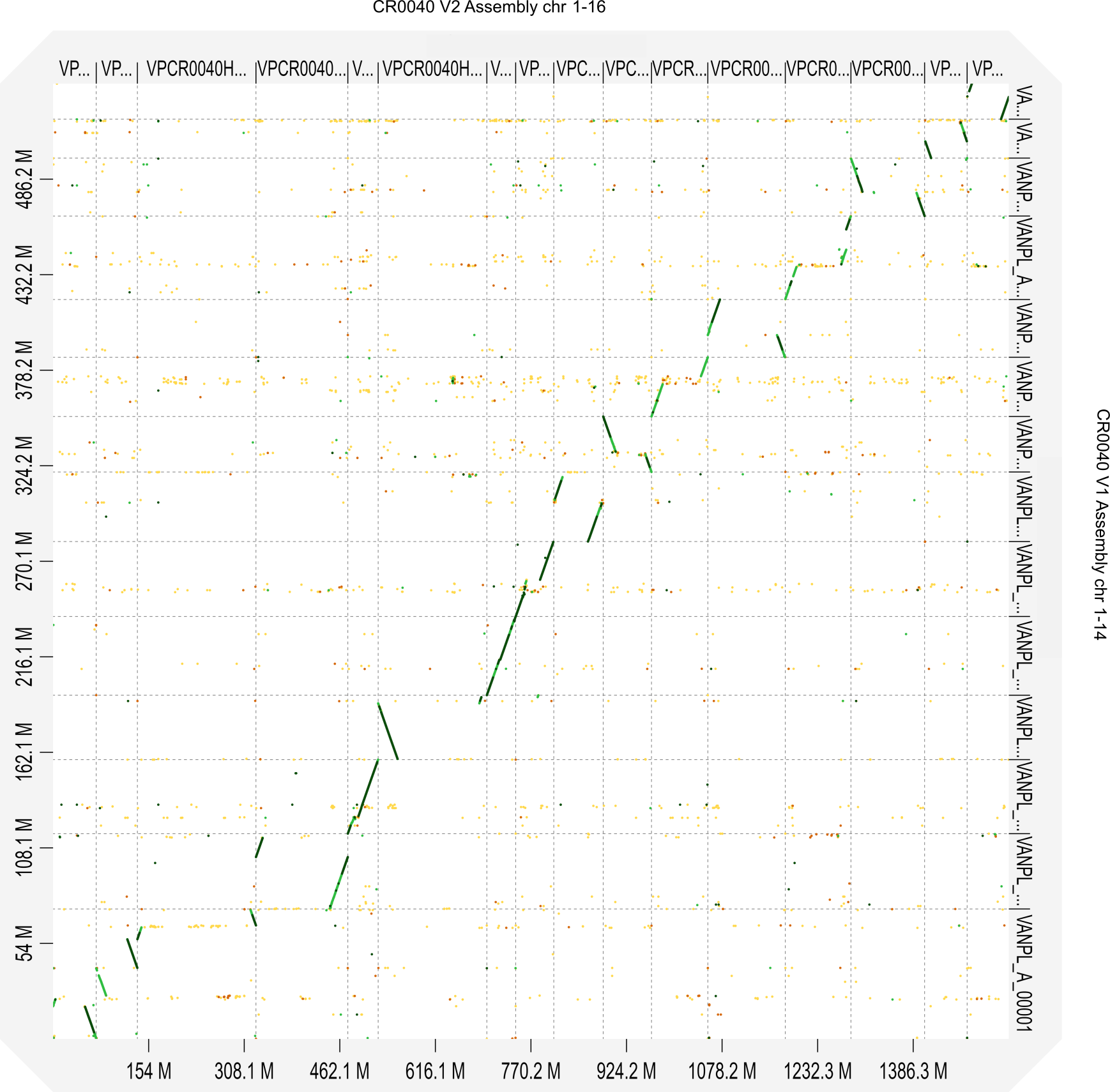


**Figure S2.** Dotplot of the alignment between the 14 chromosomes from CR0040 V1 haplotype A (y-axis) and the 16 chromosomes from CR0040 V2 haplotype A (x-axis).


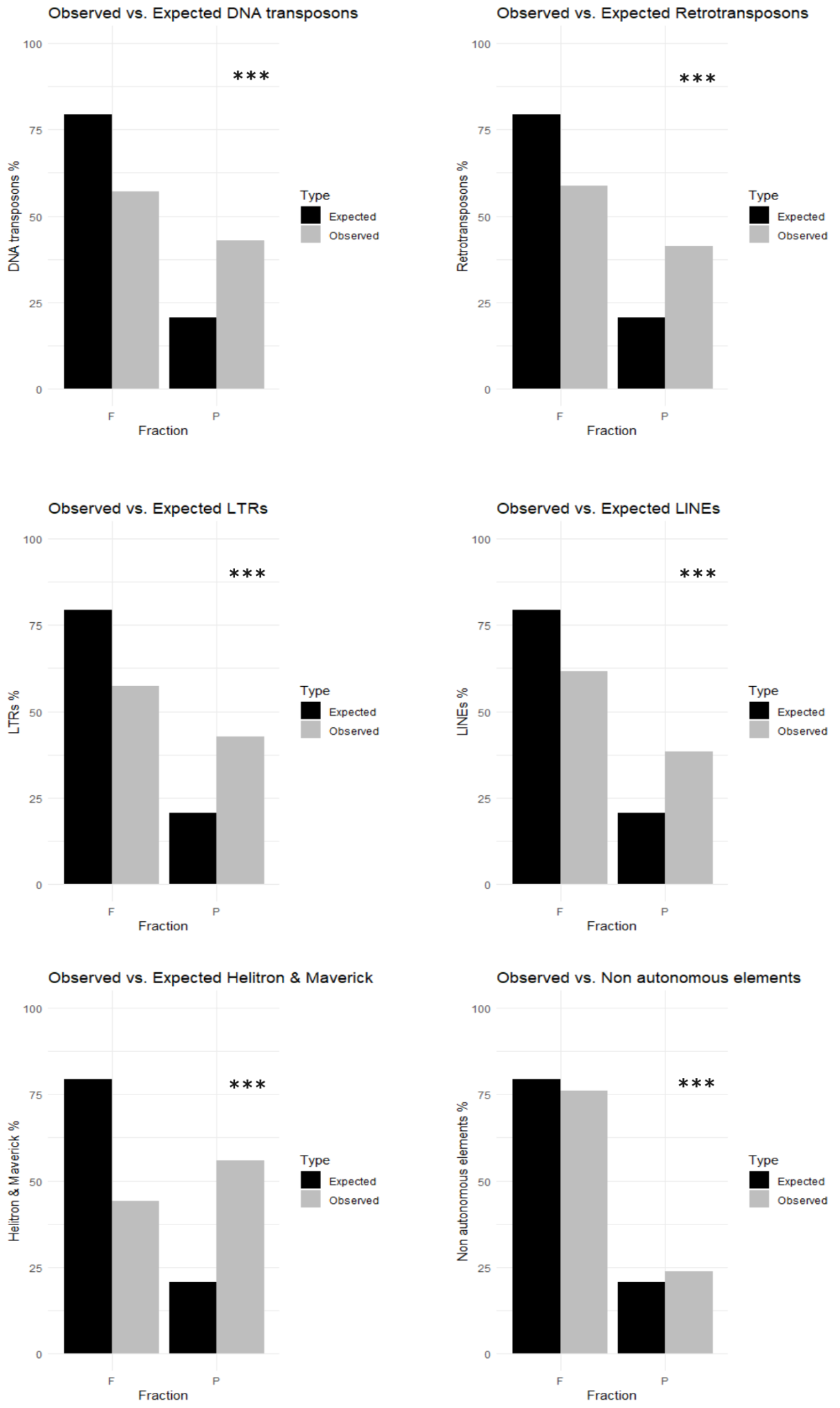


**Figure S3.** Preferential distribution of *Vanilla planifolia* transposable elements between F and P fractions across all 16 pairs of assembled chromosomes. (***) p-value < 0.001.
